# The mental health and wellbeing of care-experienced young people during early and later adolescence

**DOI:** 10.1177/13591045251333028

**Published:** 2025-04-21

**Authors:** Bethan Carter, Katherine H Shelton, Lisa J Holmes, Eva A Sprecher, Maryam Javed, John Macleod, Jeongeun Park, Julie Selwyn, Iram Siraj, Charlotte Robinson, Rachel M Hiller

**Affiliations:** 1 2112 Cardiff University, UK; 2 1948 University of Sussex, UK; 3 4919 University College London (UCL), UK; 4 1980 University of Bristol, UK; 5 6396 University of Oxford, UK; 6 University of Maynooth, Ireland

**Keywords:** Mental health, wellbeing, adoption, foster care, care-experienced, out-of-home care

## Abstract

**Background:**

Care-experienced young people (CEYP) have far higher rates of mental ill-health than their peers. Less is known about their wellbeing and the overlap between mental health and wellbeing in this population. Drawing on two samples of CEYP, we explored mental health and wellbeing profiles, the overlap between these, and basic predictors of symptom severity.

**Methods:**

We recruited two samples of CEYP: 269 10-13-year-olds and 155 16-17-year-olds, and their primary caregiver. All participants were either in local authority (out-of-home) care or had been adopted from the care system in England and Wales. Participants completed standardised measures of anxiety-, depression-, PTSD-, and externalising symptoms, as well as standardised wellbeing measures.

**Results:**

The majority of young people in both samples reported clinically-elevated symptomology, with mental health and wellbeing particularly poor in the late adolescents sample. Almost half of the 16-17 year old sample rated their wellbeing as poor. Overall, we found moderate associations between mental health and wellbeing. In early adolescents, these associations were less clear (many with clinically-elevated mental health reported average wellbeing), but for older teens poor mental health was closely related with the poorest reported wellbeing. There was no consistent evidence that age, gender, or ethnicity predicted wellbeing, but mental health was generally the poorest for older teens in residential care placements.

**Conclusions:**

We found high levels of disorder-specific mental health symptomology in CEYP, with 16–17-year-olds having particularly high levels of mental health difficulties and low wellbeing. Results highlight the crucial role of early intervention and prevention in this group, before difficulties become entrenched and affect wider aspects of wellbeing.

## Plain language summary

There is a lack of research on the mental health and wellbeing of care-experienced young people, which has an impact on the support young people receive. Our paper highlights common mental health symptoms (e.g. anxiety, depression, post-traumatic stress) among care-experienced young people over two key ages: early and late adolescence. We show that mental health and wellbeing are distinct but overlapping constructs, and that this overlap is particularly strong by late adolescence. Our findings highlight the urgent need for early intervention and preventative support for care-experienced young people, to reduce the likelihood of mental health difficulties and poor wellbeing as they approach adulthood.

## Introduction

Care-experienced young people (CEYP) often face significant early adversity and interpersonal trauma, with child maltreatment being a primary reason for their entry into state care (known as local authority care in the UK; Department for Education, 2022). Research shows CEYP experience rates of mental health difficulties approximately five times higher than their peers; around half of children in care meet the criteria for a diagnosable mental health condition (Bronsard et al., 2016; Engler et al., 2022; Ford et al., 2007). Similarly, young people adopted from local authority care are at greater risk of mental health difficulties compared to their non-adopted peers (Paine et al., 2021) and are twice as likely to engage with professional mental health services (Barroso et al., 2017; Behle & Pinquart, 2016; Keyes et al., 2008).

While we have good evidence of the mental health needs of CEYP (although this evidence is predominantly from studies published almost 20 years ago), the UK evidence base for CEYP’s wellbeing is more recently emerging, and there is limited research on the intersection of mental health and wellbeing. Anthony et al. (2022) used national Welsh school survey data and found that CEYP had lower subjective wellbeing compared to their non-care-experienced peers, with those in residential care having the lowest subjective wellbeing. Wijedasa et al. (2022) presented self-reported mental health symptoms and subjective wellbeing profiles of CEYP aged 11–18 years during the COVID-19 pandemic, compared to the general population. CEYP reported higher mental health difficulties and significantly lower wellbeing compared with the general population (Wijedasa et al., 2022). Another large-scale study, with almost 5,000 CEYP, explored demographic and service level factors associated with wellbeing and found that a longer length of time in care, fewer placement moves and being male were associated with higher wellbeing (Suh & Selwyn, 2023). Associations between mental health and wellbeing were not explored in these studies.

There is an ongoing debate regarding whether mental health and wellbeing are distinct constructs or two ends of a single spectrum. The single continuum model posits that wellbeing is the absence of mental ill-health and that enhancing wellbeing can reduce mental health difficulties, and vice versa. Conversely, the dual continua model (Keyes, 2002) contends that mental health and wellbeing are separate but related constructs, implying that individuals can experience mental health difficulties while maintaining high wellbeing (Gautam et al., 2024). Proponents of the dual continua model argue that while mental health refers to an individual’s overall psychological state which allows them to function in daily life, wellbeing is a broader concept that encompasses life satisfaction, perceived quality of life and the ability to cope with stress and adversity (Keyes, 2002). There is growing evidence in the general population of young people that mental health and wellbeing are separate but related constructs (Kinderman et al., 2015; Patalay & Fitzsimons, 2016). For example, one study found there only to be a modest association between child reported mental health and wellbeing (Sharpe et al., 2016), and another reported that mental health and wellbeing have both shared and unique predictors (Patalay and Fitzsimons (2016). It is unclear if these findings hold amongst CEYP.

We aimed to characterise the mental health and wellbeing profiles of CEYP and explore the overlap in these constructs, during early and later adolescence, drawing on two samples of CEYP (one of 10–13-year-olds and one of 16–17-year-olds). Adolescence can be a period of significant change, including in mental health and wellbeing, which is potentially even more potent in a population of young people who have high rates of trauma exposure and experience high rates of instability (Stein & Dumaret, 2011; Tarren-Sweeney, 2008). Our second aim was to explore demographic and service level predictors of mental health and wellbeing for CEYP, including age (given mental health difficulties tend to increase over adolescence; Goldbeck et al., 2007; Rice et al., 2003); sex (with evidence females are more likely to experience internalising difficulties and males more likely to display externalising behaviours; Ford et al., 2007); ethnicity (seldom explored but some evidence that White CEYP experience higher mental health difficulties compared to CEYP from other ethnic groups; Hiller et al., 2023; Wijedasa et al., 2022) and placement type (with some evidence that young people in residential care have particularly poor mental health and wellbeing; Ford et al., 2007; Hiller et al., 2021). This evidence is important for understanding the applicability of key mental health and wellbeing theories for CEYP and for providing services with higher quality evidence on the needs of their young people, including any demographic markers of risk.

## Method

### Participants

We drew on two samples of CEYP, recruited as part of the ReThink project (see https://osf.io/7qx54). One sample comprised 269 10–13-year-olds and the other sample was 155 16–17-year-olds. All young people had experience of the care system in England and Wales. The samples were mainly recruited via 13 English and Welsh local authorities, with 86% currently under local authority care (i.e., in out-of-home care). Most of these young people were living with an unrelated foster carer (67%), with an average placement length of 4.6 years (ranging from a new placement to 16.8 years). Eight-percent of the samples had been adopted from local authority care and recruited via adoption agencies. See Table 1 for detailed demographics. Exclusion criteria included the presence of a severe neurodevelopmental condition or learning difficulty which would have prevented questionnaire completion; significant current active suicidal ideation or psychosis; or an insufficient level of English language proficiency.

**Table 1.** Demographic Characteristics of the Sample.

	10–13-year-olds		16–17-year-olds	
	n	%	n	%
Demographic	Young person			
Total sample	268		155	
Sex at birth				
Male	143	53.9	58	37.4
Female	123	46.1	97	62.6
Gender				
Male	142	53.4	59	38.6
Female	118	44.4	83	54.2
Non-binary	4	1.5	7	4.6
Prefer not to say	2	0.8	4	2.6
Age (years)				
10	45	16.8	-	-
11	66	24.6	-	-
12	79	29.5	-	-
13	78	29.1	-	-
16	-	-	29	18.7
17	-	-	126	81.3
Ethnicity				
White	208	77.9	111	71.6
Black	19	7.1	13	8.4
Mixed	32	12.0	19	12.3
Asian	1	0.4	7	4.5
Other	7	2.6	5	3.2
Placement type				
Foster care	201	75.0	82	52.8
Kinship care	32	11.9	13	8.4
Residential care	10	3.7	9	5.8
Semi-independent / Independent / Supported/ Other	0	0.0	39	25.2
Unknown	0	0.0	4	2.6
Adoption	25	9.3	8	5.2
English first language				
Yes	244	93.5	140	90.3
No	17	6.5	15	9.7
Not in education, training or employment (NEET)				
Yes	-	-	21	13.7
No	-	-	132	86.3
	Caregiver			
Total sample	211		75	
Carer gender				
Male	25	11.9	12	16.0
Female	185	88.1	63	84.0
Carer ethnicity				
White	193	91.5	67	89.3
Black	13	6.2	3	4.0
Mixed	2	0.5	4	5.3
Asian	3	1.4	0	0
Other	1	0.5	1	1.3
Carer age (years)	M	SD	M	SD
	51.6	10.4	51.9	11.7

For all participants there was the option for their caregiver to participate. For consistency, we use the term caregiver to refer to any primary caregiver of the participant, whether a foster carer, kinship carer, adoptive parent, or keyworker (for those living in residential care). For the younger group, 216 (80%) caregivers participated, while for the older group, 75 (48%) caregivers participated. The lower participation in the older group was because many of the young people in local authority care reported not having a consistent and trusted adult who they would want to report on their mental health. Descriptive data for the caregivers are in Table 1.

### Procedure

We used baseline data from an ongoing longitudinal research study that is registered on the Open Science Framework [https://osf.io/7qx54]. Ethical Approval was obtained from the University College London Research Ethics Committee (Ref: 22253/001), along with approval from the Association of Directors of Children’s Services and research governance requirements of the participating Local Authorities. Safeguarding and risk escalation procedures were in place and mental health assessment summary letters were sent to social workers or (adoptive) parents when necessary.

For those in care, senior local authority staff in the thirteen participating local authorities provided informed consent for young people to participate in the research. Young people provided their own informed assent (or consent if 16+ years old). Caregivers provided informed consent for their own participation. In total, local authority consent was provided for 2,951 young people. Of those, 1,064 young people (36%) were either not contactable due to incorrect contact details (10%) or were not eligible to participate (26%; e.g., were not in the age range). Of the remaining 1,872, 421 CEYP participated (equating to 22% of those contactable and eligible). In addition, adoptive parent-child dyads were recruited via adoption networks. Here, the parent provided informed consent and young person provided informed assent (or consent if 16+ years old).

Among the 809 young people (43%) who declined participation after contact, the main reasons were young people not being interested or they stated that it was not a good time. Following receipt of consent/assent, young people and their caregiver completed questionnaires. These were either completed online via RedCap (online software), via post, over the telephone, or during an in-person home visit.

### Measures

All measures were completed by young people and their caregiver, unless specified.

#### Internalising and externalising difficulties

The 25-item Strengths and Difficulties Questionnaire (SDQ; Goodman et al., 2001) consists of two subscales each of 10 items, which measure internalising (emotion and peer problems) and externalising difficulties (conduct and hyperactivity problems). The five-item subscale for prosocial skills is not reported here. Each item is rated on a zero (not true) to 2 (certainly true) scale. Scores on each subscale are summed to give total internalising and externalising difficulties scores from 0-20, with higher scores indicative of a higher level of symptoms. Total difficulties scores are the sum of the internalising and externalising subscales, giving possible values from 0-40. Cronbach’s alpha for SDQ total difficulties was .83 (10-13-year-olds) and .86 (16-17-year-olds). The SDQ uses a four-category system to categorise internalising, externalising and total difficulty severity (close to average, slightly raised, high, very high).

#### Anxiety and depression symptoms

The Revised Child Anxiety and Depression Scale (RCADS-25; Ebesutani et al., 2017) is a 25-item self-report questionnaire, assessing anxiety and depression symptoms. The measure contains 15-items measuring anxiety symptoms (total score range = 0–45); and 10-items measuring depression symptoms (total score range = 0–30). The items are summed to give total anxiety and depression symptoms from 0-75. Each item is rated from zero (Never) to 3 (Always) with higher scores indicating higher levels of anxiety and/or depression symptoms. Cronbach’s alpha for RCADS total scores was .92 (10-13-year-olds) and .95 (16-17-year-olds). Scores can be transformed into sex and age adjusted *t-*scores and compared to clinical cut-offs based on normative datasets. These cut-offs are used to create a categorical variable with three groups: normal, at the borderline clinical threshold, and above the clinical threshold.

#### Post-traumatic stress disorder symptoms (PTSD)

The 8-item Child Revised Impact of Events Scale (CRIES-8; Perrin et al., 2005) was used to assess young people's PTSD symptomology. Items are scored on a four-point scale (0 = not at all; 1 = rarely; 3 = sometimes; 5 = often) and summed to give a total score, with scores of 17 and above indicating possible PTSD. Cronbach’s alpha for total CRIES scores was .85 (10-13-year-olds) and .88 (16-17-year-olds). A dichotomous variable was also created with scores 17 and above in the ‘above threshold’ group. The measure has good psychometric properties (Deeba et al., 2014; Verlinden et al., 2014).

#### Subjective wellbeing

The 14-item self-report Warwick-Edinburgh Mental Wellbeing Scale (WEMWBS; Tennant et al., 2007) was used to assess subjective wellbeing, including affective-emotional aspects, cognitive aspects, and psychological functioning for individuals. Items are scored on a Likert scale from 1 (none of the time) to 5 (all of the time). Total scores range between 14 and 70, with higher scores representing higher levels of wellbeing. The scale has been reported to be valid and reliable across a range of child and adolescent populations in the UK (Clarke et al., 2011; Tennant et al., 2007). Cronbach’s alpha for total subjective wellbeing scores was .87 (10-13-year-olds) and .90 (16-17-year-olds). Scores can be divided into high, average and low wellbeing using cut points at plus or minus one standard deviation based on general population samples (Tennant et al., 2007). These categories are derived from the top 15% of scores (scores of 60–70; high wellbeing) and bottom 15% of scores (scores of 14–42; poor wellbeing) from UK population normed data (Stewart-Brown & Janmohamed, 2008).

#### School satisfaction

The School Satisfaction Scale (SSS; Huebner et al., 2001) is an 8-item school subscale of the 40-item self-report Multidimensional Students’ Life Satisfaction Scale (Huebner, 1994). The SSS measures student life satisfaction across five key domains. Items are scored on a 6-point Likert scale ranging from 1 (strongly disagree) to 6 (strongly agree). Items are summed to give total scores; higher scores indicate higher levels of school satisfaction. Cronbach’s alpha was .75. School satisfaction was not measured in the older age group (16–17 years old) because many would have been in learning environments other than secondary schools (e.g. Further Education Colleges).

#### Caregiver reported health-related quality of life

Health-related quality of life was reported by carers/parents using the KINDL-R Questionnaire (KINDL; Ravens-Sieberer & Bullinger, 1998). The questionnaire consists of 24-items across six dimensions: physical wellbeing, emotional wellbeing, self-esteem, family, social contacts, and school. Items are scored on a 5-point scale ranging from 1 (never) to 5 (all the time), with higher scores indicating better quality of life. The six dimensions form six subscales with four items each. The ‘family’ subscale refers to the home the young person was living in. Cronbach’s alpha for total KINDL scores was .75 (subscales α = .65 to .88). The KINDL does not have clinical cut-off scores based on normative population data.

#### Demographics

Age, gender, and ethnicity were self-reported by both age groups. Age was measured in years; options for gender identity included boy, girl, non-binary, and prefer not to say, with a free report option if the above options did not apply. Of note, whilst only a few young people chose 'prefer not to say', many of these participants identified as gender nonconforming in the free-report text. For statistical analyses, given the small number of non-binary or ‘prefer not to say' responses (see Table 1), gender was categorised as boy (coded as 0) and girl, non-binary, or preferred not to say (coded as 1, hereon referred to as girls and non-binary for brevity). As a sensitivity check, we re-ran the analysis coded only as boys (0) and girls (1) (which excluded participants not in these categories), and there was no difference in the results. Ethnicity was collected from the following categories: White, Black, Mixed, Asian, Other. For statistical analysis, given the small number of Black, Mixed, Asian and ‘Other Ethnicity' young people, this was collapsed into White (coded as 0) and non-White (coded as 1).

#### Service level factor

Placement type was recorded as where young people were living at the time the measures were completed. Placements included non-biological foster carer, kinship foster care, residential care, semi-independent accommodation, independent accommodation, other accommodation, and adoption. For analysis, this was collapsed into (0) “family-based care” (foster, kinship, adoption), and (1) “non-family based care” (e.g., residential, semi-independent).

### Data analytic plan

Data were analysed using SPSS v25. Our primary aims were to explore (1) the mental health and wellbeing profiles of care-experienced young people during early and late adolescence, (2) to explore associations between mental health and wellbeing outcomes, and (3) to understand whether demographic and service level factors predicted mental health and wellbeing outcomes in these groups. In both age groups, self-reported anxiety and depression symptoms were positively skewed so square root transformations were applied. Among the 10–13-year-olds, carer-reported child physical wellbeing was negatively skewed, so an inverse transformation was applied. We then used bivariate correlational analyses to understand the basic associations between the mental health and wellbeing outcome variables in each age group. Despite transformation, carer-reported child physical wellbeing remained skewed. Associations were checked using non-parametric tests (Spearman’s rho), with discrepancies noted (see Table 3). Fisher *r*-to-*z* transformations were conducted to assess differences in correlation effect sizes.

Chi-square tests were used to explore whether high mental ill-health (defined as scoring above established clinical-threshold on the CRIES-8, RCADS-25, SDQ; see Methods) was associated with the poorest wellbeing (defined as the lowest 15% of WEMWBS scores, from nationally normed data (see Measures). This analysis sought to more robustly explore whether poor mental health and poor wellbeing could be considered similar in CEYP, given implications for service delivery.

Finally, we were interested in whether demographic features (age, gender, ethnicity) and service-level (placement type) variables were associated with outcomes. For the 10–13 year olds, placement type was ultimately not included as the vast majority were in a family-based placement (see Table 1). For the 16-17 year-olds, age was not included, as these data were only available as a whole year (i.e., either 16 or 17). First, we used bivariate and point biserial correlations to explore basic associations between the predictors and symptom severity, focused on child-reported outcomes (SDQ, RCADS, CRIES-8, WEMWBS scores). Where associations were significant (*p* < .05), we conducted linear regressions to explore the strongest predictors of mental health and wellbeing. The largest group was treated as the reference group (see Measures) for the binary demographic variables.

## Results

### Descriptive statistics

Among the 10–13-year-olds, 54% were male and 78% were White. Three-quarters (75%) were in foster care, with a mean current placement length (if in care) of 4.2 years (range 0–12.9 years). Among the 16-17-year-olds, 39% were male, 70% were White, and 55% were in foster care (mean current placement length = 6.3 years; range 0–16.9 years). Full sample descriptive data are reported in Table 1.

### Mental health characteristics of CEYP

There were generally high rates of mental health difficulties in both age groups. Mean scores and proportions in standardised ranges for each measure are presented in Table 2. Approximately one-third of the sample, in both age ranges, scored in the high or very high range on the SDQ (total difficulties). For the disorder-specific symptom measures rates of poor mental health were far higher in the older sample. Among the 10–13-year-olds, 5.6% were above clinical-threshold for anxiety, 5.6% for depression, while 54.3% were above threshold for PTSD symptoms. Among the 16–17-year-olds, 21.9% self-reported clinically-elevated anxiety, 27.1% depression, and 71.0% PTSD symptoms. There was a clear difference in wellbeing scores between early and later adolescence with 14% of 10–13-year-olds reporting low wellbeing versus 43% of 16–17-year-olds (see Table 2).

**Table 2.** Mental Health and Wellbeing Outcome descriptive Statistics.

	10–13-year-olds			16–17-year-olds		
	Mean	SD	Range	Mean	SD	Range
SDQ (YP report)						
Internalising	5.72	3.53	0–16	7.69	4.10	1–19
Externalising	8.07	4.14	0–19	7.40	4.15	0–19
Total	13.79	6.58	0–35	15.09	7.32	0–32
RCADS-25 (YP report)						
Anxiety	9.90	7.96	0–42	14.44	10.09	0–45
Depression	6.18	5.21	0–27	11.54	7.72	0–30
Total	16.09	12.31	0–69	25.99	16.87	0–75
CRIES-8 (YP report)						
Total score	17.29	10.61	0–40	22.18	11.35	0–40
WEMWBS (YP report)						
Total score	51.56	9.01	26–70	44.38	10.77	14–68
SSS (YP report)	4.01	0.93	1.63–5.63	-	-	-
KINDL (parent/carer report)	(*n* = 205)			(*n* = 73)		
Total	91.19	13.67	59–120	85.87	14.16	43–110
Physical wellbeing	16.68	2.75	7–20	14.65	3.74	6–20
Emotional wellbeing	16.36	2.82	8–20	15.06	3.11	6–20
Self-esteem	13.00	3.26	4–20	12.38	3.01	4–20
Family	16.00	2.86	5–20	17.00	2.62	7–20
Friends	14.29	3.36	5–20	14.04	3.48	4–20
School	14.86	3.11	6–20	12.74	2.95	6–20
						
	n	%		n	%	
SDQ Internalising^a^						
Close to average	117	43.8		54	35.5	
Slightly raised	87	32.6		56	36.8	
High	34	12.7		20	13.2	
Very high	29	10.9		22	14.5	
SDQ Externalising^a^						
Close to average	78	29.3		31	20.1	
Slightly raised	111	41.7		66	42.9	
High	30	11.3		20	13.0	
Very high	47	17.7		37	24.0	
SDQ Total^a^						
Close to average	149	55.6		78	51.3	
Slightly raised	41	15.3		24	15.8	
High	21	7.8		11	7.2	
Very high	57	21.3		39	25.7	
CRIES-8 Total^b^						
Below threshold	123	45.7		45	29.0	
Above threshold	146	54.3		110	71.0	
RCADS-25 anxiety^c^						
Normal	239	90.7		108	69.7	
Borderline	10	3.7		13	8.4	
Clinical	15	5.6		34	21.9	
RCADS-25 depression^c^						
Normal	243	92.0		105	67.7	
Borderline	7	2.7		8	5.2	
Clinical	15	5.3		42	27.1	
RCADS-25 total^c^ –						
Normal	239	90.5		100	64.5	
Borderline	12	4.5		11	7.1	
Clinical	12	4.5		44	28.4	
WEMWBS^d^						
Low	38	14.2		66	42.6	
Average	185	69.0		76	49.0	
High	45	16.8		13	8.4	

^a^Raised cut-off for SDQ total scores is 16 for child-report and 14 for carer-report, the clinical cut off is 20 for child-report and 17 for carer-report.

^b^Clinical cut-off for CRIES-8 is 17.

^c^Borderline cut-off for RCADS-25 adjusted t-scores is 65 and clinical cut-off is 70.

^d^High and low categories are the top and bottom 15% of scores based on UK general population samples.

### Associations between mental health symptoms and wellbeing

Mental health and wellbeing scores were moderately negatively correlated in both age groups, with higher mental health difficulties associated with lower wellbeing scores. The exception to this was PTSD symptoms (discussed below).

### 10–13-year-old CEYP

There was a strong, negative correlation between child self-reported subjective wellbeing and depression symptoms (*r* = −.61), and moderate negative correlations between general wellbeing and internalising difficulties (*r* = −.53), externalising difficulties (*r* = −.39), and anxiety symptoms (*r* = −.46), indicating that higher mental health difficulties were associated with lower subjective wellbeing scores. There was a weaker correlation between PTSD symptom severity and self-reported subjective wellbeing (*r* = −.15), and Fisher’s *r*-to-*z* transformations indicated that the correlation magnitude was significantly weaker than that between the other mental health outcomes and wellbeing (probability of *z*-scores less than 0.05). There were also weak correlations between child-reported mental health and child-reported school satisfaction, and PTSD symptomology was not significantly associated with school satisfaction. Child-reported mental health symptoms were significantly correlated (small to medium effects) with caregiver reported school, friendship, and ‘family relationships’ wellbeing (see Table 3).

**Table 3.** Pearson Bivariate Correlations Between Mental Health and Wellbeing Measures for 10–13-Year-olds and 16–17-Year-olds.

10–13-year-olds					
	Internalising -SDQ	Externalising - SDQ	Anxiety – RCADS-25	Depression – RCADS-25	PTSD – CRIES-8
General wellbeing - WEMWBS	−.53**	−.39**	−.46**	−.61**	−.15*
School satisfaction - SSS	−.31**	−.29**	−.19*^a^	−.39**	0.01
Physical wellbeing - KINDL	−.35**	−.16*	−.24**	−.31**	−0.05
Emotional wellbeing - KINDL	−.40**	−.28**	−.30**	−.44**	−.18*
Self-esteem - KINDL	−.29**	−.29**	−.16*^a^	−.29**	−0.13^a^
Family - KINDL	−.27**	−.42**	−.15*	−.27**	−.15*
School - KINDL	−.49**	−.35**	−.30**	−.35**	−.15*
Friends - KINDL	−.39**	−.40**	−.19**	−.33**	−0.08

16-17-year-olds					
	Internalising - SDQ	Externalising - SDQ	Anxiety – RCADS-25	Depression – RCADS-25	PTSD – CRIES-8
Wellbeing - WEMWBS	−.56**	−.50**	−.51**	−.59**	−.40**
Physical wellbeing - KINDL	−.36**	−.27*	−.37**	−.54**	−.33**
Emotional wellbeing - KINDL	−.48**	−.27**	−.37**	−.54**	−.33*
Self-esteem - KINDL	−.36**	−.37**	−.34*	−.52**	−.34**
Family - KINDL	−.25*	−.36**	−.26*	−.39**	−.17
School - KINDL	−.33**	−.19	−.12	−.22	−.37**
Friends - KINDL	−.40**	−.21	−.38**	−.37**	−.27*

*p < .05 **p < .01.

^a^Using Spearman’s Rho, at 10–13 years the strength of the association between self-reported school satisfaction and anxiety symptoms reduced from *p* < .001 to *p* < .05; and carer-reported self-esteem was less associated with self-reported anxiety and PTSD symptoms. There were no other discrepancies between parametric and non-parametric tests.

### 16-17-Year-old CEYP

For the older sample, there were moderate to strong negative correlations between subjective wellbeing and every mental health measure (*r* = −.40 to −.59), indicating that higher mental health symptom scores were associated with lower general wellbeing scores (Table 3 and supplemental material). From the caregiver report of wellbeing (*n* = 72), young person-reported mental health symptoms were significantly correlated (small to medium effects) with carer-reported ‘family relationships’, school, and friendship wellbeing, although these aspects of wellbeing were not consistently associated with all mental health measures (see Table 3 and supplemental material).

### Proportionate overlap between high mental ill health and low wellbeing

When looking at categories of mental health (above/below thresholds) and categories of wellbeing (low/poor; average; high/good), young people with clinically elevated mental health symptoms rarely rated their wellbeing as ‘high/good’. However, for 10–13-year-olds, those with high mental health need were generally spread between rating their wellbeing as ‘poor’ or ‘average’, whereas by 16-17-year-old, most of those with the poorest mental health also rated their wellbeing as poor (see Table 4).

**Table 4.** Cross Tabulations of total scores on the young Person-reported Mental Health Measures and Wellbeing Measured Using WEMWBS.

10–13-year-olds									
Wellbeing - WEMWBS									
		Low		Average		High		Total	Chi square test statistic (*p* value)
		n	%	n	%	n	%		
SDQ total	Close to average	6	4%	110	74%	33	22%	150	45.42 (*p* < .001)
	Slightly raised	5	12%	28	68%	8	20%	40	
	High	6	29%	14	62%	2	9%	22	
	Very high	21	37%	34	60%	2	3%	57	
	Total	38		187		44		269	
Anxiety & depression – RCADS-25	Normal	26	11%	171	71%	42	18%	240	30.11 (*p* < .001)
	Borderline	4	33%	7	58%	1	8%	12	
	Clinical	8	62%	5	39%	0	0%	13	
	Total	38		185		43		265	
PTSD – CRIES-8	Below threshold	12	10%	88	72%	22	18%	127	3.50 (*p* = .17)
	Above threshold	26	18%	97	66%	23	16%	150	
	Total	38		185		45		277	

16-17-year-olds									
		Low		Average		High		Total	Chi square test statistic (*p* value)
SDQ total	Close to average	13	17%	53	68%	12	15%	78	62.19 (*p* < .001)
	Slightly raised	11	46%	12	50%	1	4%	24	
	High	6	55%	5	45%	0	0%	11	
	Very high	35	90%	4	10%	0	0%	39	
	Total	65		74		13		152	
Anxiety & depression – RCADS-25	Normal	29	29%	59	59%	12	12%	100	27.97 (*p* < .001)
	Borderline	4	36%	7	64%	0	0%	11	
	Clinical	33	75%	10	23%	1	2%	44	
	Total	66		76		13		155	
PTSD – CRIES-8	Below threshold	9	20%	30	67%	6	13%	45	11.63 (*p* = .003)
	Above threshold	57	51%	46	43%	7	6%	110	
	Total	66		76		13		155	

### Demographic predictors of mental health and wellbeing

#### 10-13 year old CEYP

The only demographic variable associated with mental health was gender. Point-biserial correlations indicated that identifying as a girl or non-binary was associated with higher levels of anxiety and depression symptoms (*r*_pb_ = .13) compared to identifying as a boy (this finding remained when only comparing females and males). Alternatively, age was the only demographic variable associated with wellbeing, with older age associated with worse child-reported wellbeing scores (WEMWBS: *r* = −0.13). Ethnicity was not associated with outcomes (see supplementary materials for full results). As there was only evidence of single variables associated with outcomes, we did not run further regression analyses.

#### 16–17-year-olds

Gender and ethnicity showed small but significant correlations with self-reported wellbeing, with young people identifying as a girl or non-binary reporting poorer wellbeing compared to boys (*r*_pb_ = −.16) and non-White young people reporting better wellbeing (*r*_pb_ = .18). Placement type was weakly correlated with all mental health outcomes but not wellbeing. CEYP in non-family style care reported higher internalising and externalising symptoms (*r*_pb_ = .25), anxiety and depression (*r*_pb_ = .22), and higher PTSD symptoms (*r*_pb_ = .20). Gender was weakly associated with internalising and externalising difficulties (*r*_pb_ = −.23) and anxiety and depression (*r*_pb_ = .18), with girls and non-binary young people reporting higher mental health symptoms than boys.

In the regression models including gender, ethnicity and placement in a single step, gender remained a unique predictor of mental health symptoms but not wellbeing. Being a girl or non-binary was associated with higher reported internalising and externalising difficulties (*β* = 1.77, *p* = .03) and anxiety and depression (*β* = 0.24, *p* < .01) compared to boys. Ethnicity remained a unique predictor of both mental health and wellbeing, with non-White young people reporting fewer internalising and externalising difficulties (*β* = −0.18, *p* = .02) and higher reported wellbeing (*β* = 0.16, *p* = .04) compared to White young people. Placement type also remained a significant unique predictor of mental health only, with non-family based care associated with higher reporting of internalising and externalising difficulties (*β* = 0.25, *p* < .01), anxiety and depression (*β* = 0.22, *p* < .01) and PTSD symptoms (*β* = 0.20, *p* = .02).

## Discussion

We aimed to characterise the mental health and wellbeing profiles of CEYP during early and late adolescence, explore associations between mental health and wellbeing outcomes, and examine whether demographic and service level factors predicted both mental health and wellbeing. We found high levels of mental ill-health and noticeably higher levels in later adolescence. Findings also provided nuanced insight into overlaps between mental health and wellbeing, but limited consistent evidence that demographic or placement factors were robustly associated with symptom scores.

There is a paucity of evidence on the mental health and wellbeing needs of CEYP in the UK, beyond the annually reported SDQ (DfE, 2022). What is available consistently shows increased mental ill-health and lower wellbeing of CEYP, compared to the general population (Ford et al., 2007; Wijedasa et al., 2022). Here, we provide further evidence for the disorder-specific symptom profiles and wellbeing needs of CEYP and demonstrate that need is greater in later adolescence. Self-reported wellbeing was particularly poor in our older sample, suggesting a faster trajectory of worsening wellbeing than might be expected in the general population of older teens (Clarke et al., 2011; Morey et al., 2016; Sadler et al., 2018). That is, 42% of the 16–17-year-olds scored themselves in a poor wellbeing range, which would be expected of only 15% of a normed population. These findings highlight the urgency of early mental health intervention and prevention approaches for CEYP to address mental health before it worsens, or to prevent difficulties developing in later adolescence.

There is growing evidence that CEYP face additional barriers to accessing best-evidenced mental health support in a timely manner (e.g., Hiller et al., 2021; McGuire et al., 2022; McGuire et al., 2024; Phillips et al., 2024). CEYP who are 16-17-year-old are often facing other complex challenges not faced by their non-care-experienced peers, whilst also navigating the transition into early adulthood – particularly uncertainty about their living situation and a dramatic change in support and services available as they ‘age-out’ of the care-system (Stein, 2019; Stubbs et al., 2023). Almost 40% of our older sample were in non-family style placements, such as residential care homes or semi-independent placements. Reasons for being in these types of placements can be complex and sometimes this is the right type of placement for the young person’s needs (Cameron-Mathiassen et al., 2022; Holmes et al., 2018). However, these types of placements were associated with worse mental health and wellbeing in 16-17-year-olds. This likely reflects a cycle of worsening mental health, placement breakdowns, and a move to non-family care, which is widely highlighted in the literature on CEYP (Konijn et al., 2019; Maguire et al., 2024), further reflecting the urgency of high-quality, timely mental health care for CEYP. Our findings show that demographic factors are generally not reliable markers of mental health and wellbeing outcomes. For the younger sample (10-13-year-olds), being older and identifying as a girl were associated with slightly worse reported mental health, although not on all measures. For the older sample, being a girl and being White were both associated with slightly worse reported mental health, although not on all measures.

A key focus of our work was understanding the overlap between mental health and wellbeing. We explored this to understand the relevance of current theories on mental health and wellbeing for CEYP, but also because increasingly research suggests that services (particularly children’s social care) consider these concepts to be relatively unique, with implications for service development (e.g., developing wellbeing services that do not provide direct mental health support; McGuire et al., 2024). There is mounting evidence in the general population that mental health and wellbeing are separate but related constructs (Kinderman et al., 2015; Patalay & Fitzsimons, 2016). Our findings also show that mental health symptoms and wellbeing of CEYP are separate but related constructs, in line with the dual continua model (Keyes, 2005). This held true for self-report and for carer-report on multiple aspects of child wellbeing. However, there were some differences by age group. For the younger sample, while mental health and wellbeing were correlated (except for PTSD symptoms) many young people reporting clinically elevated mental ill-health self-reported average wellbeing (although few reported ‘good’ wellbeing). By late adolescence, this was much rarer and there was a stronger overlap between these constructs (e.g., among those who scored in the clinical range for anxiety and depression, 75% also rated their wellbeing as poor). This suggests that the overlap between mental health and wellbeing sits within a developmental context, reflecting an interplay between biological changes, psychological factors (e.g., worsening mental health corroding wellbeing), social pressures that can characterise adolescence (Cilar Budler & Stiglic, 2023; Goldbeck et al., 2007; Patalay & Fitzsimons, 2018; Yoon et al., 2023), and pressures unique to older adolescence in care or with care-experience (previously highlighted). Findings emphasise the need to consider CEYP’s needs holistically, including mental health and wellbeing together. Furthermore, there is a window where CEYP might be able to maintain relatively good wellbeing, which diminishes as they approach later adolescence.

## Strengths and limitations

There are few primary research projects on CEYP, and even fewer including self-report on mental health and wellbeing (usually mental health is reported by caregivers or social workers). This means we have comparatively little information on their mental health needs, compared to other populations of youth. Despite the many complex challenges of recruiting this population, it is crucial that we develop a stronger evidence-base of needs to support service decision-making. Nevertheless, findings should be considered in light of limitations. Most of the sample were recruited via local authorities, which requires complex consenting procedures. Only 22% of potentially eligible young people ultimately participated. Thus, we cannot guarantee that this is a fully representative sample of CEYP, although SDQ scores are comparable to national data published by the Department for Education (with approximately 40% scoring in the borderline or high range). Caregiver report was only available for 46% (*n* = 72) of the older sample, reflecting that many young people perceived that there was not a trusted adult who they wanted reporting on their mental health. Next, we have used standardised screening tools not diagnostic tools so we cannot say whether or not a child met full diagnostic criteria (although this was not our research aim). We also used clinical cut-offs that have predominantly been developed on non-CEYP populations. The CRIES-8 PTSD screening tool has a cut-off only validated with children who have experienced one-off traumas, but available literature suggests a lower (not higher) cut-off might be more appropriate for CEYP (Tarren-Sweeney, 2019). Additionally, the WEMWBS cut-offs are based on total general population norms, not young people specifically, and therefore should be interpreted with some caution. Nevertheless, our mean WEMWBS scores were similar to other studies with adolescents, albeit lower for the older age group (Clarke et al., 2011; Morey et al., 2016). Finally, we intentionally reported on the samples separately as we wanted to understand mental health during key transition periods (i.e., primary and early secondary school; emerging adulthood). This has provided interesting information on the different levels of needs and circumstances (e.g., placements) experienced by these two age ranges. However, we have not statistically compared these samples. These two smaller samples also meant that we were unable to explore ethnicity in a more meaningful way than collapsing into a binary (White vs. non-White) variable. Future research should aim to collect sufficient numbers of participants from ethnic minorities to enable a more nuanced analysis.

## Conclusion

We found high levels of disorder-specific mental health symptomology in CEYP. By exploring this in two distinct age ranges, we demonstrated the importance of early intervention or prevention, with a strong contrast apparent between the age groups. Almost three-quarters of 16–17-year-olds reported clinically high PTSD symptoms, and almost half of 16-17-year-old CEYP reported poor wellbeing. We also found moderate overlap between mental health and wellbeing, with these constructs particularly aligned by 16-years-old. Our findings highlight the urgent need for high quality support for the mental health and wellbeing of CEYP, so all CEYP can thrive as they move through adolescence and into adulthood.

## Supplemental Material

Supplemental Material - The mental health and wellbeing of care-experienced young people during early and later adolescence Supplemental Material for The mental health and wellbeing of care-experienced young people during early and later adolescence by Bethan Carter, Katherine H Shelton, Lisa J Holmes, Eva A Sprecher, Maryam Javed, John McLeod, Jeongeun Park, Julie Selwyn, Iram Siraj, Charlotte Robinson and Rachel M Hiller in Clinical Child Psychology and Psychiatry

## Data Availability

Analytic code, data and research materials are available on request to the corresponding author. Data presented here are, in part, baseline data from an ongoing longitudinal project (pre-registered) and will be available via UK Data Service at the conclusion of the full project*.

## Appendix

### Abbreviations

CEYP: Care-experienced young people

## References

[bibr1-13591045251333028] Anthony R. Paine A. Westlake M. Lowthian E. Shelton K. (2022). Patterns of adversity and post-traumatic stress among children adopted from care. Child Abuse & Neglect, 130(Pt 2), Article 104795. 10.1016/j.chiabu.2020.104795 33172646

[bibr2-13591045251333028] Barroso R. Barbosa-Ducharne M. Coelho V. Costa I. Silva A. (2017). Psychological adjustment in intercountry and domestic adopted adolescents: A systematic review. Child and Adolescent Social Work Journal, 34(5), 399–418. 10.1007/s10560-016-0485-x

[bibr3-13591045251333028] Behle A. E. Pinquart M. (2016). Psychiatric disorders and treatment in adoptees: A meta-analytic comparison with non-adoptees. Adoption Quarterly, 19(4), 284–306. 10.1080/10926755.2016.1201708

[bibr4-13591045251333028] Bronsard G. Alessandrini M. Fond G. Loundou A. Auquier P. Tordjman S. Boyer L. (2016). The prevalence of mental disorders among children and adolescents in the child Welfare system: A systematic review and meta-analysis. Medicine (Baltimore), 95(7), Article e2622. 10.1097/md.0000000000002622 26886603 PMC4998603

[bibr5-13591045251333028] Cameron-Mathiassen J. Leiper J. Simpson J. McDermott E. (2022). What was care like for me? A systematic review of the experiences of young people living in residential care. Children and Youth Services Review, 138(5), Article 106524. 10.1016/j.childyouth.2022.106524

[bibr6-13591045251333028] Cilar Budler L. Stiglic G. (2023). Age, quality of life and mental well-being in adolescent population: A network model tree analysis. Scientific Reports, 13(1), Article 17667. 10.1038/s41598-023-44493-w 37848537 PMC10582244

[bibr7-13591045251333028] Clarke A. Friede T. Putz R. Ashdown J. Martin S. Blake A. Adi Y. Parkinson J. Flynn P. Platt S. Stewart-Brown S. (2011). Warwick-Edinburgh mental well-being scale (WEMWBS): Validated for teenage school students in England and Scotland. A mixed methods assessment. BMC Public Health, 11(7), 1–9. 10.1186/1471-2458-11-487 21693055 PMC3141456

[bibr8-13591045251333028] Deeba F. Rapee R. M. Prvan T. (2014). Psychometric properties of the children’s revised Impact of events scale (CRIES) with Bangladeshi children and adolescents. PeerJ, 2(1), Article e536. 10.7717/peerj.536 25237597 PMC4157240

[bibr9-13591045251333028] Department for Education . (2022, November). *Who are the children entering care in England? *Department for Health and Social Care, Office of National Statistics. Available from. https://www.ons.gov.uk/peoplepopulationandcommunity/healthandsocialcare/socialcare/articles/whoarethechildrenenteringcareinengland/2022-11-04

[bibr10-13591045251333028] Ebesutani C. Korathu-Larson P. Nakamura B. J. Higa-McMillan C. Chorpita B. (2017). The revised child anxiety and depression scale 25–parent version: Scale development and validation in a school-based and clinical sample. Assessment, 24(6), 712–728. 10.1177/1073191115627012 26834091

[bibr11-13591045251333028] Engler A. D. Sarpong K. O. Van Horne B. S. Greeley C. S. Keefe R. J. (2022). A systematic review of mental health disorders of children in foster care. Trauma, Violence, & Abuse, 23(1), 255–264. 10.1177/1524838020941197 32686611

[bibr12-13591045251333028] Ford T. Vostanis P. Meltzer H. Goodman R. (2007). Psychiatric disorder among British children looked after by local authorities: Comparison with children living in private households. The British journal of psychiatry: The Journal of Mental Science, 190(1), 319–325. 10.1192/bjp.bp.106.025023 17401038

[bibr52-13591045251333028] Gautam S Jain A Chaudhary J Gautam M Gaur M Grover S , et al. (2024). Concept of mental health and mental well-being, it’s determinants and coping strategies. Indian Journal of Psychiatry, 66(2), S231–S244. 10.4103/indianjpsychiatry.indianjpsychiatry_707_23 38445271 PMC10911315

[bibr13-13591045251333028] Goldbeck L. Schmitz T. G. Besier T. Herschbach P. Henrich G. (2007). Life satisfaction decreases during adolescence. Quality of Life Research: An International Journal of Quality of Life Aspects of Treatment, Care and Rehabilitation, 16(6), 969–979. 10.1007/s11136-007-9205-5 17440827

[bibr14-13591045251333028] Goodman R. (2001). Psychometric properties of the strengths and difficulties questionnaire. Journal of the American Academy of Child & Adolescent Psychiatry, 40(11), 1337–1345. 10.1097/00004583-200111000-00015 11699809

[bibr15-13591045251333028] Hiller R. M. Fraser A. Denne M. Bauer A. Halligan S. L. (2023). The development of young peoples’ internalising and externalising difficulties over the first three-years in the public care system. Child Maltreatment, 28(1), 141–151. 10.1177/10775595211070765 35081783 PMC9716486

[bibr16-13591045251333028] Hiller R. M. Meiser-Stedman R. Elliott E. Banting R. Halligan S. L. (2021). A longitudinal study of cognitive predictors of (complex) post-traumatic stress in young people in out-of-home care. The Journal of Child Psychology and Psychiatry and Allied Disciplines, 62(1), 48–57. 10.1111/jcpp.13232 32196661

[bibr18-13591045251333028] Holmes L. Connolly C. Mortimer E. Hevesi R. (2018). Residential group care as a last resort: Challenging the rhetoric. Residential Treatment for Children & Youth, 35(3), 209–224. 10.1080/0886571X.2018.1455562

[bibr19-13591045251333028] Huebner E. S. (1994). Preliminary development and validation of a multidimensional life satisfaction scale for children. Psychological Assessment, 6(2), 149–158. 10.1037//1040-3590.6.2.149

[bibr20-13591045251333028] Huebner E. S. Ash C. Laughlin J. E. (2001). Life experiences, locus of control, and school satisfaction in adolescence. Social Indicators Research, 55(2), 167–183. 10.1023/A:1010939912548

[bibr21-13591045251333028] Keyes C. L. (2005). Mental illness and/or mental health? Investigating axioms of the complete state model of health. Journal of Consulting and Clinical Psychology, 73(3), 539–548. 10.1037/0022-006X.73.3.539 15982151

[bibr22-13591045251333028] Keyes C. L. M. (2002). The mental health continuum: From languishing to flourishing in life. Journal of Health and Social Behavior, 43(2), 207–222. 10.2307/3090197 12096700

[bibr23-13591045251333028] Keyes M. A. Sharma A. Elkins I. J. Iacono W. G. McGue M. (2008). The mental health of US adolescents adopted in infancy. Archives of Pediatrics and Adolescent Medicine, 162(5), 419–425. 10.1001/archpedi.162.5.419 18458187 PMC4475346

[bibr24-13591045251333028] Kinderman P. Tai S. Pontin E. Schwannauer M. Jarman I. Lisboa P. (2015). Causal and mediating factors for anxiety, depression and well-being. The British journal of psychiatry: The Journal of Mental Science, 206(6), 456–460. 10.1192/bjp.bp.114.147553 25858180

[bibr25-13591045251333028] Konijn C. Admiraal S. Baart J. Van Rooij F. Stams G.-J. Colonnesi C. Lindauer R. Assink M. (2019). Foster care placement instability: A meta-analytic review. Children and Youth Services Review, 96(1), 483–499. 10.1016/j.childyouth.2018.12.002

[bibr26-13591045251333028] Maguire D. May K. McCormack D. Fosker T. (2024). A systematic review of the Impact of placement instability on emotional and Behavioural outcomes among children in foster care. Journal of Child & Adolescent Trauma, 17(2), 641–655. 10.1007/s40653-023-00606-1 38938940 PMC11199447

[bibr28-13591045251333028] McGuire R. Halligan S. L. Meiser‐Stedman R. Durbin L. Hiller R. M. (2022). Differences in the diagnosis and treatment decisions for children in care compared to their peers: An experimental study on post‐traumatic stress disorder. British Journal of Clinical Psychology, 61(4), 1075–1088. 10.1111/bjc.12379 35702815 PMC9796033

[bibr29-13591045251333028] McGuire R. Meiser‐Stedman R. Smith P. Schmidt D. Bjornstad G. Bosworth R. Clarke T. Coombes J. Geijer Simpson E. Hudson K. (2024). Access to best‐evidenced mental health support for care‐experienced young people: Learnings from the implementation of cognitive therapy for PTSD. British Journal of Clinical Psychology, 64, 63–85. 10.1111/bjc.12471 39012021 PMC11797148

[bibr31-13591045251333028] Morey Y. Mellon D. Dailami N. Verne J. Tapp A. (2016). Adolescent self-harm in the community: An update on prevalence using a self-report survey of adolescents aged 13–18 in England. Journal of Public Health, 39(1), 58–64. 10.1093/pubmed/fdw010 26892623

[bibr32-13591045251333028] Paine A. L. Fahey K. Anthony R. E. Shelton K. H. (2021). Early adversity predicts adoptees’ enduring emotional and behavioral problems in childhood. European Child & Adolescent Psychiatry, 30(5), 721–732. 10.1007/s00787-020-01553-0 32468437 PMC8060221

[bibr33-13591045251333028] Patalay P. Fitzsimons E. (2016). Correlates of mental illness and wellbeing in children: Are they the Same? Results from the UK Millennium Cohort study. Journal of the American Academy of Child & Adolescent Psychiatry, 55(9), 771–783. 10.1016/j.jaac.2016.05.019 27566118

[bibr34-13591045251333028] Patalay P. Fitzsimons E. (2018). Development and predictors of mental ill-health and wellbeing from childhood to adolescence. Social Psychiatry and Psychiatric Epidemiology, 53(12), 1311–1323. 10.1007/s00127-018-1604-0 30259056

[bibr35-13591045251333028] Perrin S. Meiser-Stedman R. Smith P. (2005). The Children’s revised Impact of event scale (CRIES): Validity as a screening instrument for PTSD. Behavioural and Cognitive Psychotherapy, 33(4), 487–498. 10.1017/S1352465805002419

[bibr36-13591045251333028] Phillips A. R. Hiller R. M. Halligan S. L. Lavi I. Macleod J. A. Wilkins D. (2024). A qualitative investigation into care‐leavers’ experiences of accessing mental health support. Psychology and psychotherapy, 97(3), 439–455. 10.1111/papt.12525 38456637

[bibr37-13591045251333028] Ravens-Sieberer U. Bullinger M. (1998). Assessing health-related quality of life in chronically ill children with the German KINDL: First psychometric and content analytical results. Quality of Life Research: An International Journal of Quality of Life Aspects of Treatment, Care and Rehabilitation, 7(5), 399–407. 10.1023/A:1008853819715 9691720

[bibr38-13591045251333028] Rice F. Harold G. T. Thapar A. (2003). Negative life events as an account of age‐related differences in the genetic aetiology of depression in childhood and adolescence. The Journal of Child Psychology and Psychiatry and Allied Disciplines, 44(7), 977–987. 10.1111/1469-7610.00182 14531580

[bibr39-13591045251333028] Sadler K. Vizard T. Ford T. Goodman A. Goodman R. McManus S. (2018). Mental health of children and young people in England, 2017: Trends and characteristics. NHS Digital.

[bibr53-13591045251333028] Sharpe H. Patalay P. Fink E. Vostanis P. Deighton J. Wolpert M. (2016). Exploring the relationship between quality of life and mental health problems in children: implications for measurement and practice. European Child and Adolescent Psychiatry, 25(6), 659–667. 10.1007/s00787-015-0774-5 26498932

[bibr41-13591045251333028] Stein M. (2019). Supporting young people from care to adulthood: International practice. Child & Family Social Work, 24(3), 400–405. 10.1111/cfs.12473

[bibr42-13591045251333028] Stein M. Dumaret A. C. (2011). The mental health of young people aging out of care and entering adulthood: Exploring the evidence from England and France. Children and Youth Services Review, 33(12), 2504–2511. 10.1016/j.childyouth.2011.08.029

[bibr43-13591045251333028] Stewart-Brown S. Janmohamed K. (2008). Warwick-Edinburgh mental well-being scale. User guide. Version, 1(10.1037), 2. Available from. https://www.mentalhealthpromotion.net/resources/user-guide.pdf

[bibr44-13591045251333028] Stubbs A. Baidawi S. Mendes P. (2023). Young people transitioning from out-of-home care: Their experience of informal support. A scoping review. Children and Youth Services Review, 144, Article 106735. 10.1016/j.childyouth.2022.106735

[bibr45-13591045251333028] Suh E. Selwyn J. (2023). Exploring local authority variation in looked after young people’s subjective well-being. British Journal of Social Work, 53(1), 177–197. 10.1093/bjsw/bcac117

[bibr46-13591045251333028] Tarren-Sweeney M. (2008). Retrospective and concurrent predictors of the mental health of children in care. Children and Youth Services Review, 30(1), 1–25. 10.1016/j.childyouth.2007.05.014

[bibr47-13591045251333028] Tarren-Sweeney M. (2019). Do adolescents in care systematically under-report their mental health difficulties in population studies? A narrative review. Developmental Child Welfare, 1(3), 251–272. 10.1177/2516103219829483

[bibr48-13591045251333028] Tennant R. Hiller L. Fishwick R. Platt S. Joseph S. Weich S. Parkinson J. Secker J. Stewart-Brown S. (2007). The Warwick-Edinburgh mental well-being scale (WEMWBS): Development and UK validation. Health and Quality of Life Outcomes, 5(1), 1–13. 10.1186/1477-7525-5-63 18042300 PMC2222612

[bibr49-13591045251333028] Verlinden E. van Meijel E. P. Opmeer B. C. Beer R. de Roos C. Bicanic I. A. Lamers‐Winkelman F. Olff M. Boer F. Lindauer R. J. (2014). Characteristics of the Children’s revised Impact of event scale in a clinically referred Dutch sample. Journal of Traumatic Stress, 27(3), 338–344. 10.1002/jts.21910 24797017

[bibr50-13591045251333028] Wijedasa D. N. Yoon Y. Schmits F. Harding S. Hahn R. (2022). *A survey of the mental health of children and young people* . In care in England in 2020 and 2021. University of Bristol. https://mhcat.blogs.bristol.ac.uk/publications/

[bibr51-13591045251333028] Yoon Y. Eisenstadt M. Lereya S. T. Deighton J. (2023). Gender difference in the change of adolescents’ mental health and subjective wellbeing trajectories. European Child & Adolescent Psychiatry, 32(9), 1569–1578. 10.1007/s00787-022-01961-4 35246720 PMC8896070

