# Supplementary material for: The mental health and wellbeing of care-experienced young people during early and later adolescence
Source: Clin Child Psychol Psychiatry. 2025 Apr 21;30(3):611–31. doi: 10.1177/13591045251333028 (PMC12179413; doi:10.1177/13591045251333028)
Supplement: Supplemental Material - The mental health and wellbeing of care-experienced young people during early and later adolescence [file sj-pdf-1-ccp-10.1177_13591045251333028.pdf]

Table S1. Spearman's rho correlations between mental health and wellbeing measures for 10–13-year-olds and 16–17-year-olds.

**10–13-year-olds**					
	Internalising -SDQ	Externalising - SDQ	Anxiety – RCADS-25	Depression – RCADS-25	PTSD – CRIES-8
General wellbeing - WEMWBS	-.51**	-.39**	-.47**	-.59**	-.16*
School satisfaction - SSS	-.32**	-.28**	-.21**	-.37**	-0.02
Physical Well-being - KINDL	-.32**	-.15*	-.21**	-.24**	-0.02
Emotional Well-being - KINDL	-.38**	-.25**	-.32**	-.40**	-.17*
Self-esteem - KINDL	-.31**	-.29**	-.25**	-.30**	-0.18*
Family - KINDL	-.28**	-.39**	-.18*	-.27**	-.14*
School - KINDL	-.48**	-.36**	-.30**	-.33**	-.11
Friends - KINDL	-.41**	-.41**	-.24**	-.31**	-0.12
**16-17-year-olds**					
	Internalising - SDQ	Externalising - SDQ	Anxiety – RCADS-25	Depression – RCADS-25	PTSD – CRIES-8
Wellbeing - WEMWBS	-.55**	-.50**	-.47**	-.60**	-.40**
Physical Well-being - KINDL	-.30**	-.26*	-.33**	-.54**	-.32**
Emotional Well-being - KINDL	-.44**	-.24**	-.44**	-.60**	-.28*

Self-esteem - KINDL	-.36**	-.34**	-.30*	-.50**	-.28*
Family - KINDL	-.29*	-.30*	-.27*	-.35**	-.14
School - KINDL	-.34**	-.23	-.17	-.29*	-.34**
Friends - KINDL	-.35**	-.14	-.35**	-.34**	-.21

* $p < 0.05$ ** $p < 0.01$
